# Time trends of colorectal cancer incidence and associated lifestyle factors in South Korea

**DOI:** 10.1038/s41598-021-81877-2

**Published:** 2021-01-28

**Authors:** Hayeong Khil, Sung Min Kim, SungEun Hong, Hyeon Min Gil, Eugene Cheon, Dong Hoon Lee, Young Ae Kim, NaNa Keum

**Affiliations:** 1 Department of Food Science and Biotechnology, Dongguk University Graduate School, Gyeonggi, 10325 South Korea; 2 Department of Biomedical Sciences, Seoul National University Graduate School, Seoul, 03080 South Korea; 3 Department of Food Science and Biotechnology, Dongguk University, Gyeonggi, 10325 South Korea; 4 Department of Nutrition, Harvard T.H. Chan School of Public Health, Boston, MA 02138 USA; 5 Division of Cancer Control & Policy, National Cancer Center, Gyeonggi, 10408 South Korea

**Keywords:** Cancer, Oncology

## Abstract

Worldwide, South Korea had the second highest incidence rates of colorectal cancer (CRC) in 2018. To inform public health policy to prevent CRC, we aimed to identify major modifiable lifestyle factors underlying the alarming increase in CRC incidence. We obtained information on CRC statistics from the Korea National Cancer Incidence Database and on the distribution of dietary and lifestyle factors known to modify CRC risk from the Korea National Health and Nutrition Examination Survey. To examine time trends between 2001 and 2013, we calculated annual percent changes of CRC incidence rates and of prevalence of etiologic factors by sex and age. Across all sex and age groups, the most commonly diagnosed cancer was rectal cancer while the most rapidly increasing cancer was distal colon cancer. For the lifestyle factors examined, decreases in exercise were observed across all age groups of both sexes. Yet, obesity and alcoholic drinks appear more relevant CRC contributor to men, smoking to women aged 30–49 years, and processed meat intake to adults aged 30–49 years. The heterogeneous results suggest that dietary and lifestyle target to prevent CRC be tailored by sex and age.

## Introduction

In recent decades, South Korea has experienced a rapid increase in colorectal cancer (CRC) incidence rates. Worldwide, South Korea had the second highest incidence rate of CRC in 2018, with the rate estimated to be 44.5 cases per 100,000 persons per year^1^. In South Korea, the leading cause of death is cancer^2^, of which CRC death ranked third after lung and liver cancer deaths^3^. With CRC imposing a large burden on human health both globally and locally, identification of etiologic factors driving a rise in CRC incidence rates in South Korea has important implications for public health.

Development of CRC is largely attributable to the constellation of dietary and lifestyle factors associated with westernization such as poor diet characterized by high intakes of red and processed meat and alcoholic drinks and low intakes of fiber-rich foods, low physical activity, and obesity^4^. Of note, accumulating evidence suggests that etiology of CRC may be heterogeneous by anatomical subsite of cancer (e.g., proximal colon, distal colon, and rectum), with a risk factor associated possibly more strongly with a subsite than with the other subsites^5^. For example, obesity is a stronger risk factor for colon cancer than for rectal cancer^6^. Thus, populations that have undergone varying degree of changes in the prevalence of CRC risk factors show differential distribution of CRC cases by anatomical subsite^5^. In the United States, which has a greater obesity prevalence, proximal colon cancer is the most common CRC subsite^7^, while in South Korea rectal cancer accounts for the highest proportion^5^.

Considering that major risk factors driving increases in CRC incidence rates could differ across countries, public health targets to curb the rising CRC should prioritize etiologic factors based on their prevalence and time trends within the population. Therefore, to identify predominant factors underlying the escalating incidence rates of CRC in South Korea, we conducted an ecological study by examining temporal trends in CRC incidence rates in relation to concurrent changes in the distribution of dietary and lifestyle factors established to be implicated in colorectal carcinogenesis. Additionally, to address potential etiologic heterogeneity by sex and age at CRC diagnosis^5^, we conducted the analyses separately for men and women and for early-onset and late-onset CRC.

## Results

**Figure 1:**
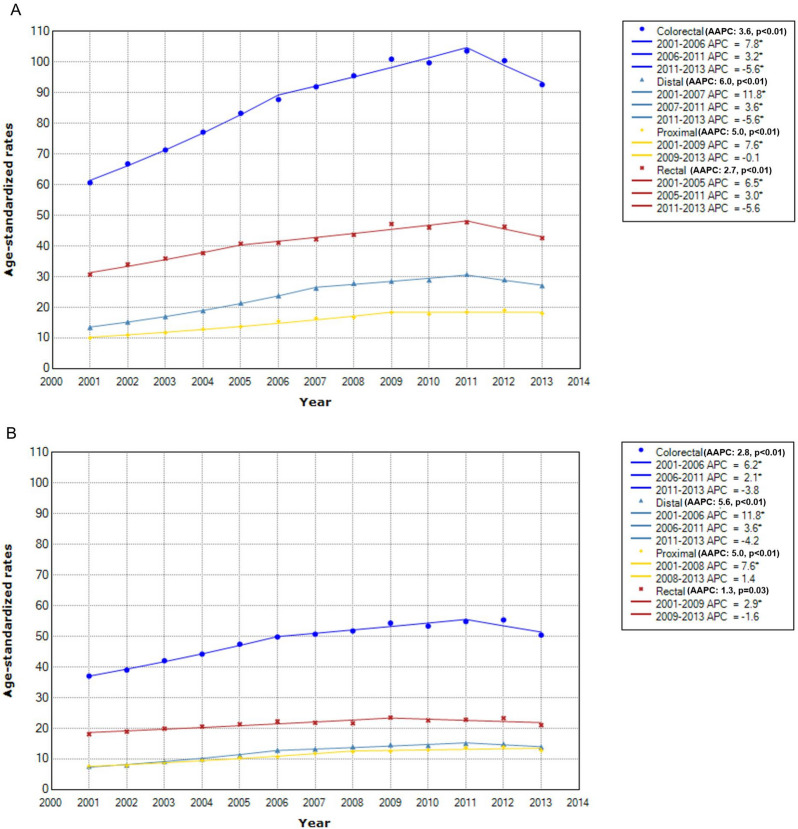
Time trend of colorectal cancer incidence rates by sex and anatomical subsite. *Indicates that APC is significantly different from zero at the alpha = 0.05 level. (**A**) In men. (**B**) In women.

CRC incidence rates showed a significantly increasing trend from 2001 to 2011 in men and women (Fig. 1). For this interval, ASR per 100,000 persons per year increased from 60.7 to 103.6 in men (Fig. 1A) and from 37.1 to 50.4 in women (Fig. 1B). However, during 2011 through 2013, the rates started to decline especially in men with an annual pace of − 5.6% (p = 0.03) (Fig. 1A). On average, the AAPC of the entire 2001–2013 period was positive in men (3.6%, p < 0.01) and women (2.8%, p < 0.01), indicating that the trend of CRC incidence rates were overall upward.

By anatomical subsite of CRC, in both men and women, the most commonly diagnosed cancer was rectal cancer, but the degree of annual increase in incidence rates was highest for distal colon cancer, followed by proximal colon cancer and rectal cancer (Fig. 1). The AAPC of the 2001–2013 period for distal colon, proximal colon, and rectal cancer was respectively 6.0 (p < 0.01), 5.0 (p < 0.01), and 2.7 (p < 0.01) in men (Fig. 1A) and 5.6 (p < 0.01), 5.0 (p < 0.01), and 1.3 (p < 0.01) in women (Fig. 1B). Despite the overall increasing trend, incidence rates of distal colon and rectal cancer through 2011 to 2013 trend showed a downward trend in men, with APC of − 5.6% (p < 0.01) and − 5.6% (p = 0.11), respectively (Fig. 1A).

**Figure 2:**
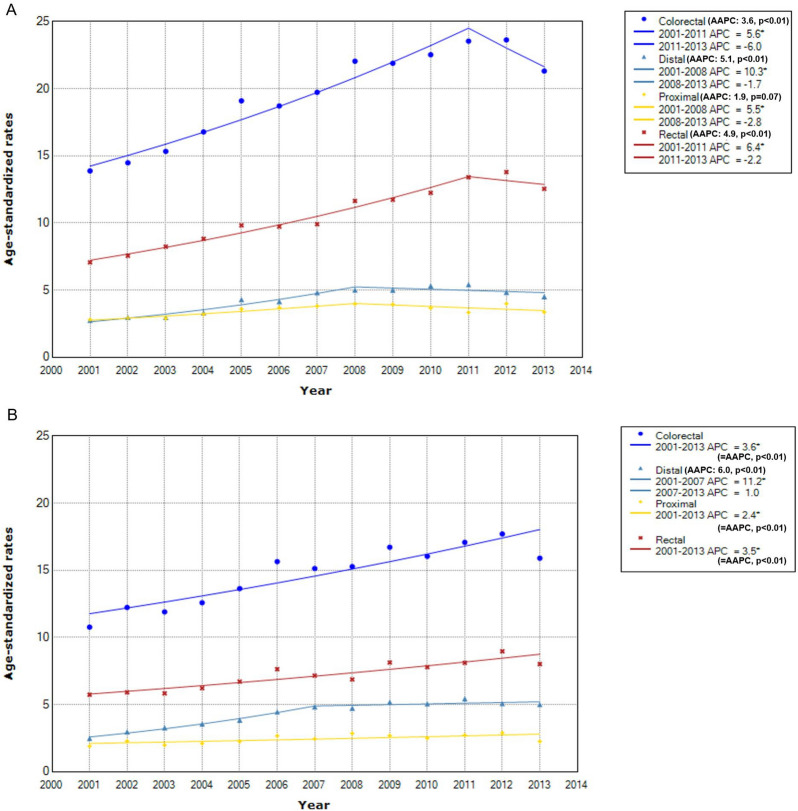
Time trend of early-onset colorectal cancer incidence rates by sex and anatomical subsite. * Indicates that APC is significantly different from zero at the alpha = 0.05 level. (**A**) In men. (**B**) In women.

**Figure 3:**
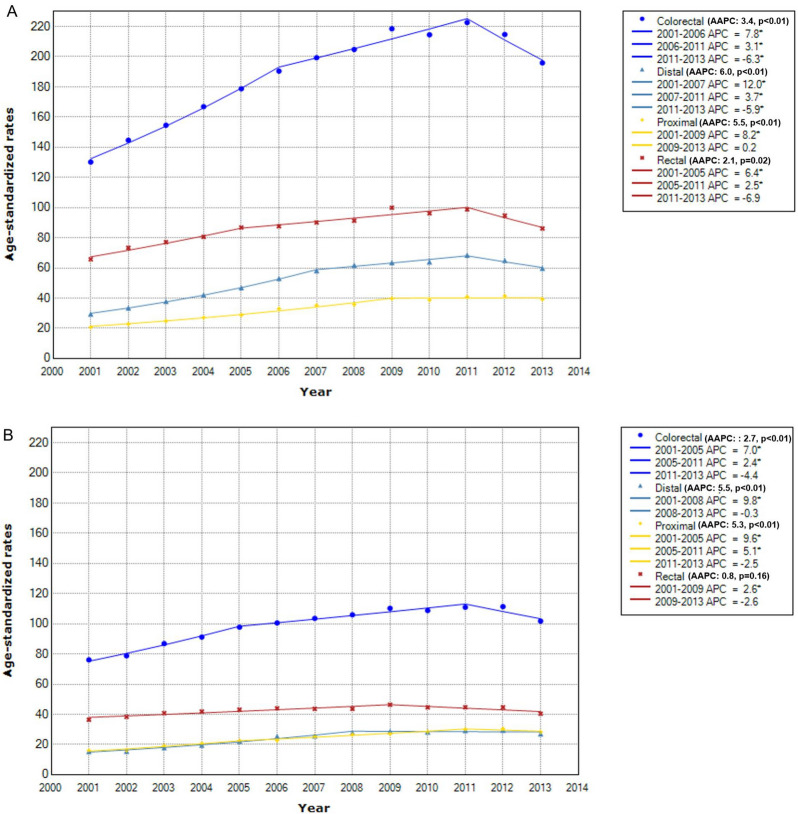
Time trend of late-onset colorectal cancer incidence rates by sex and anatomical subsite. *Indicates that APC is significantly different from zero at the alpha = 0.05 level. (**A**) In men. (**B**) In women.

By age of cancer diagnosis, the absolute levels of incidence rates were much lower for early-onset CRC (e.g., in 2011, ASR for early-onset vs. late-onset was 23.6 vs. 222.7 per 100,000 in men; 17.1 vs. 111.0 in women). Yet, the degree of annual increase on average was higher for early-onset CRC, with AAPC of the 2001–2013 interval for early-onset vs. late-onset being 3.6% (p < 0.01) vs. 3.4% (p < 0.01) in men (Figs. 2A, 3A) and 3.6% (p < 0.01) vs. 2.7% (p < 0.01) (Figs. 2B, 3B) in women. For both early-onset and late-onset CRC, the sudden downturn of CRC incidence rates during the 2011–2013 periods was more evident in men. The APC for men vs. women was − 6.0% (p = 0.26) vs. 3.6% (p < 0.01) for early-onset CRC (Fig. 2) and − 6.3% (p = 0.03) vs. − 4.4% (p = 0.07) for late-onset CRC (Fig. 3).

For both early-onset (Fig. 2) and late-onset CRC (Fig. 3), rectal cancer ranked the highest in incidence rates, but distal colon cancer experienced the largest annual increase. Yet, the gap in AAPC between distal and proximal colon cancer was much smaller for late-onset case than for early-onset case, which was more notable in women (0.2% difference for late-onset vs. 3.6% difference for early-onset) (Figs. 2B, 3B) than in men (0.5% difference for late-onset vs. 3.2% difference for early-onset) (Figs. 2A, 3A). When late-onset CRC was grouped further finely according to age of cancer diagnosis (Supplementary Figure S1, S2), in both men and women, the highest AAPC was observed with distal colon cancer between age of 50–69 years (Supplementary Figure S1a-S1b, S2a-S2b) but shifted to proximal colon cancer since age of 70 years (Supplementary Figure S1c-S1d, S2c-S2d). Specifically, in women, not only AAPC but also ASR for proximal colon cancer exceeded ASR for distal colon cancer (Supplementary Figure S2c-S2d).

**Figure 4:**
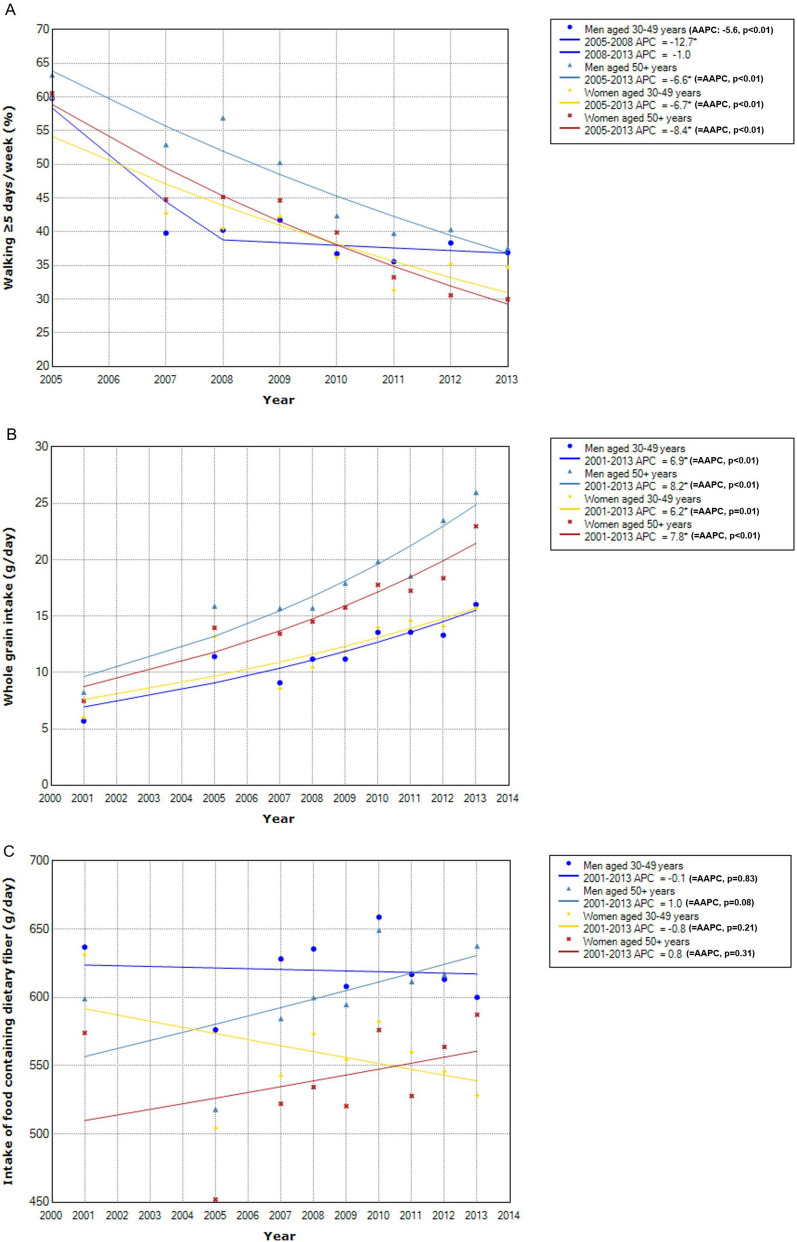

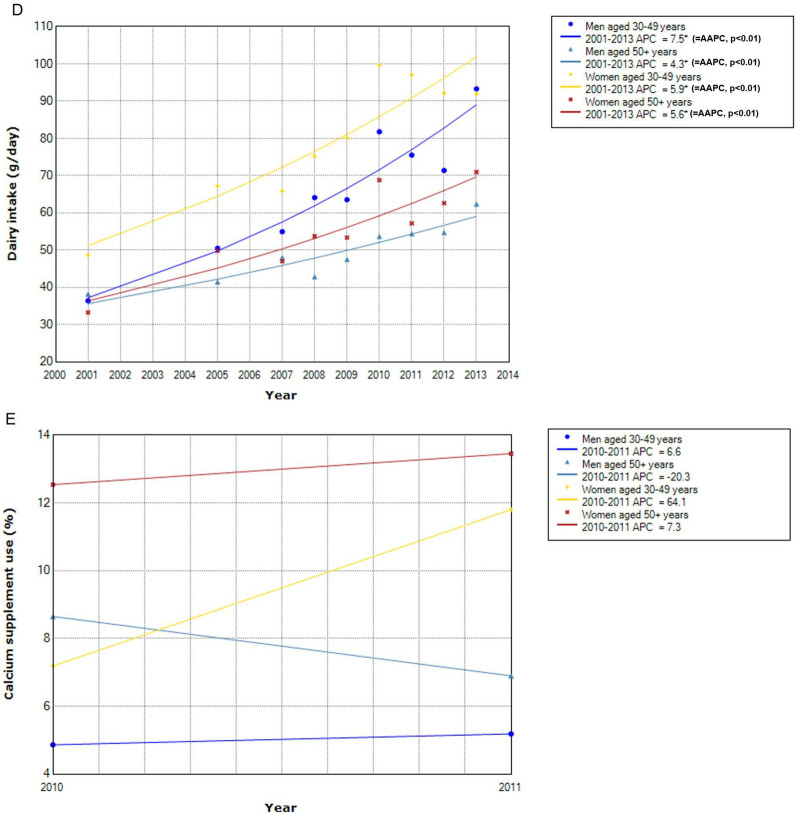
Time trend of distribution of protective factors by sex and age. * Indicates that APC is significantly different from zero at the alpha = 0.05 level. (**A**) Physical activity (walking at least 5 days per week). (**B**) Whole grain intake. (**C**) Intake of food containing dietary fiber. (**D**) Dairy intake. (**E**) Calcium supplement use.

Concerning the time trend of distributions of known CRC protective factors (Fig. 4), the absolute levels were generally higher in men than in women in later adulthood except for intakes of dairy products and calcium supplement. The direction of change in the distribution of each factor over time occurred consistently regardless of sex and age. The level of physical activity decreased significantly in all groups (Fig. 4A). Whole grain intake increased significantly in all groups (Fig. 4B). Intake of fiber-rich foods did not change significantly over time (Fig. 4C). For dairy products, the intake increased significantly in all groups (Fig. 4D). The proportion of South Koreans who took calcium supplements in 2010–2011 was higher in women than men and higher in later adulthood than early adulthood within women (12.5 vs. 7.2% in 2010; 13.5% vs. 11.8% in 2011) and men (8.7% vs. 4.9% in 2010; 7.2% vs. 5.2% in 2011) (Fig. 4E).

**Figure 5:**
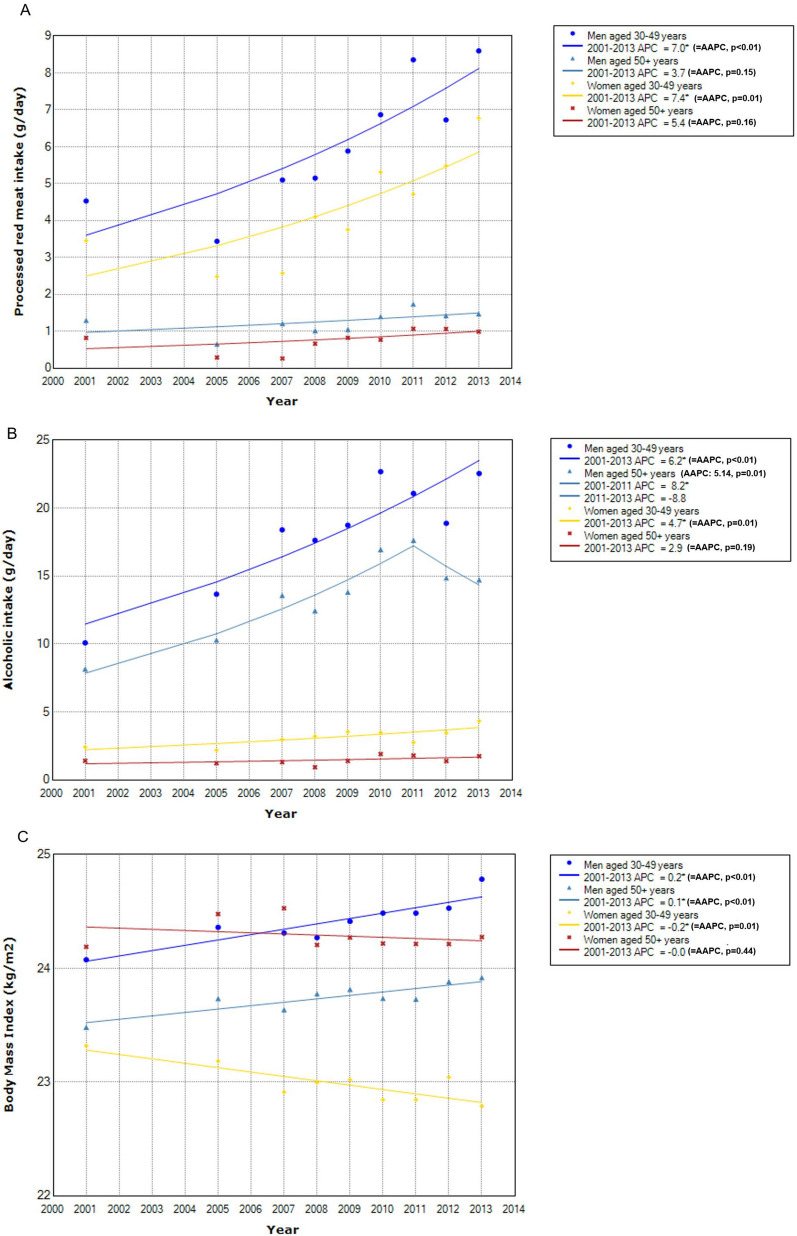

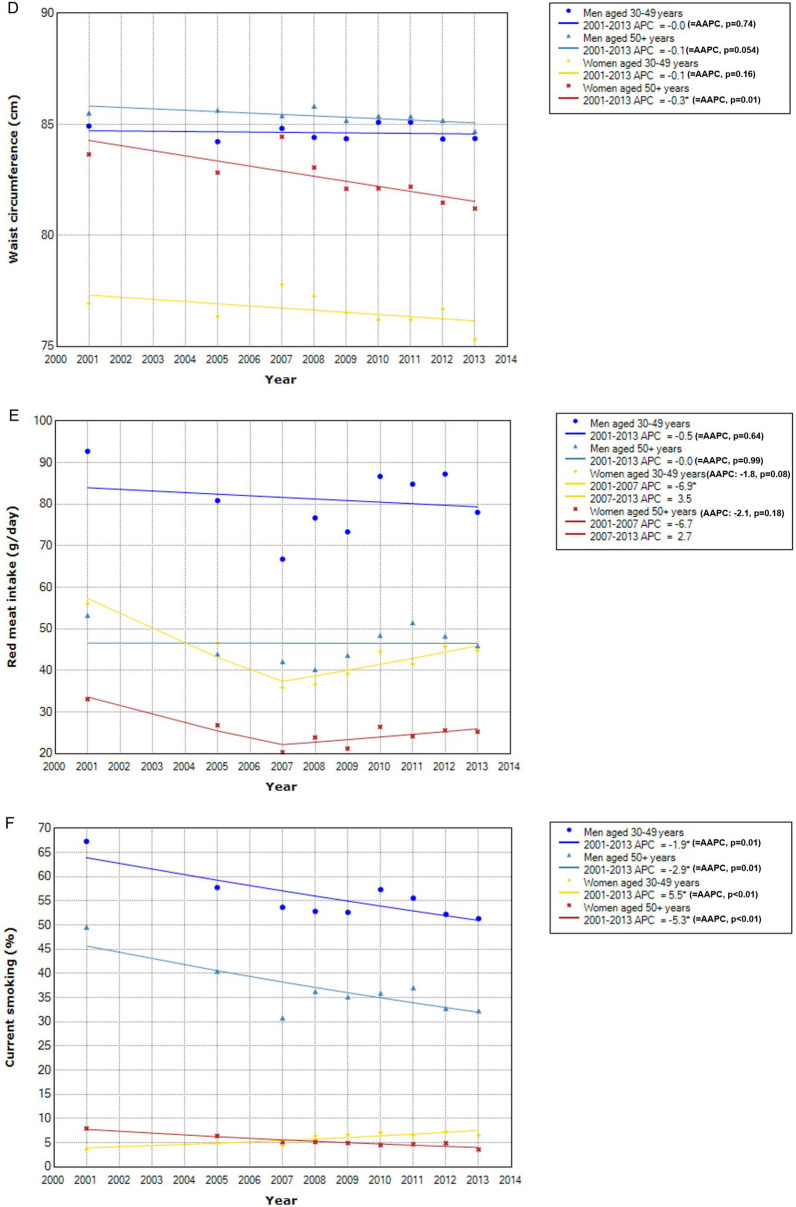
Time trend of distribution of risk factors by sex and age. *Indicates that APC is significantly different from zero at the alpha = 0.05 level. (**A**) Processed red meat intake. (**B**) Alcoholic intake. (**C**) Body Mass Index. (**D**) Waist circumference. (**E**) Red meat intake. (**F**) Current smoking.

For the time trend of distribution of known CRC risk factors (Fig. 5), the absolute levels were generally higher in men than in women across the age groups except for body fatness, for which women in later adulthood had higher BMI. Temporal change in the distribution of each factor varied by sex and age groups. Processed meat intake increased significantly in middle-aged men and women but not in those in later adulthood (Fig. 5A). Alcohol intake increased significantly except for women in later adulthood, with the degree of increase more pronounced in men than in women (Fig. 5B). For body fatness, overall adiposity as indicated by BMI increased significantly in men of all age groups but decreased significantly in middle-aged women (Fig. 5C). By abdominal adiposity as measured by waist circumference, a significant decrease was observed in women in later adulthood (Fig. 5D). Overall, no significant changes were indicated for red meat intake, although a significant decrease between 2001 and 2007 was observed for middle-aged women (Fig. 5E). The prevalence of current smokers decreased significantly in all groups but middle-aged women, among whom the prevalence increased significantly (Fig. 5F).

## Discussion

In this study that aims to identify factors associated with a rapid increase in CRC incidence in South Korea in recent past, we examined secular changes from 2001 to 2013 in CRC incidence rates and in the distribution of diet and lifestyle factors known to be associated with CRC risk. In all groups of sex (men, women) and age (30–49, 50 + years), rectal cancer was the most commonly diagnosed cancer but distal colon cancer had the largest annual % increase on average. Yet, concurrent changes in the distribution of known CRC etiologic factors were heterogeneous by sex and age groups. In men, those aged 30–49 years showed decreases in physical activity and increases in intakes of processed meat and alcoholic beverages, and BMI; those aged 50 years or older experienced decreases in physical activity and increases in alcohol consumption, and BMI. In women, those aged 30–49 years showed decreases in physical activity and increases in intakes of processed meat and alcohol beverages and smoking; those aged 50 years or older experienced decreases in physical activity for women aged 50 years or older. Despite the overall upward trends in CRC incidence rates and in the prevalence of the aforementioned etiologic factors, a significant downturn in CRC incidence rates from 2011 through 2013 was observed especially in men.

In our study, decreases in physical activity were a common change that occurred along with increases in CRC incidence across all age and sex groups. Although ecologic study design precludes causal inference, there are numerous biological mechanisms that suggest a role for physical activity in colorectal carcinogenesis. Physical activity helps reduce exposure time of intestinal mucosa to carcinogens including secondary bile acid^8–10^. Physical activity has beneficial effect against obesity and diabetes, which are established risk factors for CRC^5,11,12^. In the Health Professionals follow-up study that examined the risk of digestive system cancer by type and intensity of physical activity, aerobic exercise, regardless of its intensity, was found to be particularly beneficial, with the optimal benefit observed at approximately 30 MET-hours/week^5,13^. Walking is the most popular form of aerobic exercise worldwide and the amount of walking South Koreans engaging in has been suboptimal and decreased over time, as shown in our results.

In South Korea, higher CRC rates were observed in men than in women and this may relate to higher obesity and alcohol drinking in men. Men, particularly Asian men, have tendency toward visceral obesity, which has been suggested as the underlying player linking obesity and CRC^14^. Regarding alcohol drinking, in a recent meta-analysis of cohort studies, light (5–≤ 15 g/day) and moderate (> 15–≤ 30 g/day) drinking were significantly associated with 6% and 19% increased CRC risk, respectively, in men, but not in women^15^. The differential association by sex may be in part explained by higher level of alcohol intake in men. In South Korea, from 2001 to 2013, absolute alcohol consumption in men was high and also rapidly increased from 8.5 to 19.4 g/day. In women, on the contrary, the absolute intake remained very low (< 5 g/day) and increased only minimally (from 1.1 to 3.3 g/day) over the same time period (Fig. 5B). In a pooled analysis of 8 cohort studies, very light (> 0–< 5 g/day) alcohol consumption was not associated with CRC risk compared to no alcohol consumption^16^.

Among South Korean adults aged 30–49 years, increases in processed meat consumption was notable, which may have contributed to increases in early-onset CRC. By the International Agency for Research on Cancer Monograph Working Group, processed meat has been classified as “carcinogenic to humans” (Group 1)^17^. Likely carcinogenic compounds of processed meat include N-nitroso compounds (NOCs), which are added as preservatives or formed endogenously in the gastrointestinal through the reaction across nitrite, amines, and amides from processed meat^17,18^. The endogenous formation of NOCs by processed meats is further facilitated by heme–iron in red meat^19,20^. NOCs act as genotoxins inducing DNA mutations and thus promoting carcinogenesis^19–22^.

Unlike the distribution of CRC in high incidence countries^5,7,23^, rectal cancer was more prevalent than colon cancer in South Korea. Smoking may be a potential contributor, considering its heterogeneous associations by anatomical subsite of the colorectum and lagged effect. Smoking was generally more strongly associated with rectal cancer (RR 1.19, 95% CI 0.94–1.54) than colon cancer (RR 1.10, 95% CI 0.89–1.36)^5^. Of note, the effect of smoking on rectal cancer outcomes emerged with a long latent period^5^. For instance, in a large prospective study of US veterans, RR of rectal cancer mortality comparing current smokers versus nonsmokers was only 1.1 after 16 years of follow-up^24^, but increased to 1.4 upon 26 years of follow-up^25^. The long latency suggests that higher prevalence of rectal cancer observed in South Korea in recent years may be associated with increases in smoking in distant past. Indeed, there was a rapid growth in the annual consumption of cigarettes in South Korea from the 1970s to 1980s (from 39,672 to 69,899 million cigarettes)^26,27^.

Rapid increase in distal colon cancer was notable in the epidemiology of CRC in South Korea. A potential explanation might relate to low calcium intake in South Korea. Considerable evidence suggests that the benefit of calcium intake against CRC might be confined to primarily distal colon cancer but generally less so to cancers in other segments of the colorectum^4,28–31^. Among South Koreans, the average calcium intake from food sources is low (approximately 507 mg/day^32^ vs. 1000–1200 mg/day recommend dietary allowance of calcium for adults^33^) kimchi and tofu, in which calcium has lower bioavailability than dairy products, constitute a large part of the intake^32^. In addition, calcium supplements are not widely used (< 15%). According to a dose–response meta-analysis of prospective observational studies, each 300 mg/day increase in total calcium intake (from both food and supplemental sources) was associated with an approximately 8% reduced risk of CRC^34^. The association was observed well beyond 1000 mg/day of calcium intake^32^, which suggests that efforts to increase calcium intake might help curb the rising incidence of distal colon cancer in South Korea. Of note, given evidence that calcium helps reduce visceral adiposity^35^ calcium may confer additional benefit in preventing CRC among men in South Korea, for whom visceral fat was suggested as an important contributor to rising incidence of CRC.

Despite the overall upward trends in CRC incidence rates over the 2001–2013 period, a sudden downturn in the rates from 2011 through 2013 was evident in men. In light of concurrent declines in distal and rectal cancer rates during the 2011–2013 interval among men in later adulthood, screening effect may provide a potential explanation. In South Korea, since 2005, the National Cancer Screening Program has provided individuals aged 50 years or older with CRC screening at no or minimal cost biennially until 2011 and then annually from 2012^36^. Individuals with positive results from fecal occult blood test, the primary modality for mass screening, are entitled to either endoscopy or double-contrast barium enema^36^. From 2005 to 2008, the proportion of South Koreans who received endoscopy screening increased from 18.0 to 20.5%^37^. Of note, distal colon cancer predominantly occurs in men than in women, and it is readily detected by endoscopy due to its tendency toward polypoid morphology rather than flat morphology, which is frequently found in proximal colon cancer^38^. Furthermore, in South Korea, men were associated with a higher endoscopy uptake than women^37^. Taken together, endoscopy screening may have conferred a greater protection against CRC in men, which manifested as a downward trend in CRC incidence rates in the 2011–2013 interval. The time lag between the introduction of the nationwide CRC screening program and decreases in CRC incidence relates to the initial rise in cancer detection due to increasing screening^39^. In view of the highest annual % increase in distal colon cancer in South Korea and greater effectiveness of endoscopy in detecting distal colon cancer than proximal colon cancer, efforts to increase participation rates in CRC screening program may be of particular importance to lower CRC incidence in South Korea.

Our study has major weaknesses that limit the establishment of causal relationships. First, as an ecological study based on population-level averages, any association observed between changes in the level of lifestyle factors and changes in CRC incidence cannot be ascribed to associations between individual-level characteristics. Second, while colorectal carcinogenesis spans several decades, our study juxtaposed temporal trends over the same specified period due to lack of national statistics on the lifestyle factors in earlier periods. Thus, our study could not address temporality or potential lag-time between change in lifestyle factors and change in CRC risk. However, only few factors such as folate, calcium, and smoking have been found to act early on colorectal carcinogenesis^5,40^, and our study did not intend to estimate relative risk between a specific factor and CRC risk but rather examined consistency or inconsistency of temporal trend between multiple established etiologic factors and CRC incidence. Other concern in our study includes measurement error in the dietary data. The 24-hour recall is generally considered reliable for estimating population average intake due to cancellation of random errors across individuals. Yet, until before 2007, the dietary data in South Korea were collected at a specific season rather than throughout the year, failing to account for seasonal variability. This, along with changes in food/nutrition composition data over time, might have generated systematic errors in estimating average dietary intake.

Yet, our study has several strengths. As a country with the second highest CRC incidence rates in the world, an investigation to identify major contributing factors to this epidemic furthers our understanding of colorectal carcinogenesis. Given sex difference in CRC risk^41^ and recent increase in early-onset CRC^42,43^, our study to identify major contributors to CRC specific to sex and age groups provides a scientific basis for tailored public health intervention strategy for effective CRC prevention. Finally, statistics on CRC incidence are accurate because CRC cases were ascertained from the South Korea Central Cancer Registry, a nationwide cancer database, which captures over 90% of incidence cancer cases in South Korea^44^.

In conclusion, patterns in CRC incidence and major etiologic contributors may be highly heterogeneous by sex and age in South Korea. While decreasing physical activity appears to be a common driver of increasing CRC across all sex and age groups of South Koreans, obesity and alcoholic drinks may be more relevant CRC contributors to men, and processed meat to middle-aged adults. Efforts to increase calcium intake and CRC screening uptake may help control increasing distal colon cancer in South Korea. Our study provides some supporting evidence for tailored public health campaigns to reduce CRC, necessitating more rigorous epidemiologic studies than ecologic study on this research topic.

## Methods

### Data sources

Information on the number of CRC cases, anatomical subsites, date of diagnosis, and sex was obtained from the Korea National Cancer Incidence Database, sourced from a nationwide hospital-based cancer registry constructed by the Korea Central Cancer Registry^45^. Based on the codes of the 10th revision of the International Statistical Classification of Diseases and Related Health Problems^46^, CRC was defined as C18-C20; and further classified by anatomical subsite into proximal colon cancer (C18.0–18.4), distal colon cancer (C18.5–18.7), and rectal cancer (C19–20)^32^. Colon cancer cases coded as C18.8 (overlapping lesions of the colon) or C18.9 (colon not otherwise specified) were excluded (n = 20,873 cases, 7.6%) from the subsite analysis. National population statistics were obtained from Korean Statistical Information Service by the Ministry of the Interior and Safety^47^.

Established etiologic factors of CRC that are modifiable, such as risk factors (processed meat, alcoholic drinks, body fatness, red meat, and smoking) or protective factors (physical activity, whole grains, foods containing dietary fiber, dairy products, and calcium supplements), were selected based on the 2017 extensive summary report by World Cancer Research Fund (WCRF) and American Institute of Cancer Research (AICR)^4^.

Annual distributions of these dietary and lifestyle factors in South Korea were estimated based on responses from the Korea National Health and Nutrition Examination Survey, a series of nationally representative cross-sectional surveys conducted by the Korea Disease Control and Prevention Agency^48–50^. To elaborate on this further, dietary data were obtained from 24-hour diet recall. Processed meat, defined as any red meats preserved by smoking, salting, curing, or adding other chemical preservatives, included bacon, ham, sausage, etc. Red meat included beef, pork, lamb, goat from domesticated animals^4^. Alcoholic drinks included beer, soju (South Korean distilled spirits), makgeolli (South Korean rice wine). Whole grains included brown rice, barely, pearl barley, sorghum, oat, rye, buckwheat, millet, etc. Foods containing dietary fiber included whole grains, fruits, vegetables, and legumes. Dairy products included milk and any foods made from milk such as cheese and yogurt. Body fatness were indicated by body mass index (BMI) and waist circumference. Obesity was defined as BMI ≥ 25 kg/m^2^. Physical activity was estimated by the proportion of individuals who walked a total of ≥ 30 minutes per day (in bouts of at least 10 minutes\ duration) at least 5 days per week. Smoking was estimated by the proportion of current smokers with lifetime smoking of at least 100 cigarettes.

### Statistical analysis

For each pre-specified group of age at diagnosis (30–39, 40–49, 50–59, 60–69, 70–79, 80 +), annual incidence rates of cancer (new cases per 100,000 individuals at risk per year) were calculated for the entire colorectum and by subsites from 2001 to 2013. For cases diagnosed over a wider age range (i.e., total CRCs diagnosed at 30–80 + years of age, early-onset CRC diagnosed at 30–49 years of age, and late-onset CRC diagnosed at age of 50 + years), in order to compare annual incidence rates from 2001 to 2013 without being affected by changes in population age structure over time, age-standardized rates (ASRs) were calculated through direct standardization by averaging age-specific incidence rates weighing to mid-year age structure of South Korean population in 2005, the recommended standard population by Statistics Korea^49^.

Time trends of CRC incidence rates and distributions of aforementioned etiologic factors were described using the Joinpoint Regression Program, Version 4.8.0.1 (available at: https://surveillance.cancer.gov/joinpoint/) provided by the U.S. National Cancer Institute. In brief, joinpoint regression analysis fits a series of joined straight lines to the trend data (i.e., ASR or prevalence of etiologic factors over time), wherein each joinpoint indicates a statistically significant change in linear slope (i.e., trend)^51^. In finding the best fitting joinpoint model, we allowed a maximum of 2 joinpoints and calculated the annual percent change (APC) (i.e., slope) in ASR and prevalence for each line segment and the average annual percent change (AAPC) (i.e., average of slopes) for the whole period. The AAPC, as a weighted average of APCs with weights equal to the length of each line segment, quantifies the average trend over the whole period accounting for changes in trends during the interval^51^. The unit for APC and AAPC were expressed as % change per year. Of note, information on calcium supplement use was available from 2010 and thus, only the prevalence was estimated.

CRC incidence rates and their APCs were calculated by anatomic sites, age at diagnosis, and sex; APCs of etiologic factors were calculated by age groups and sex. All statistical tests were two-sided, and P values < 0.05 were considered statistically significant. All statistical analyses were performed using SAS 9.3 (SAS Institute, Cary, NC).

## Supplementary Information

Supplementary Information

## Data Availability

The Korea National Health and Nutrition Examination Survey (KNHANES) data are available from the Korea Centers for Disease Control and Prevention database through the following URLs: https://knhanes.cdc.go.kr/knhanes/sub03/sub03_02_02.do However, data downloads and guidebook for users are only available in South Korean language. The Korea National Cancer Incidence Database is not publicly available and only researchers who got a permission from National Cancer Center can use it.
