# Supplementary material for: Time trends of colorectal cancer incidence and associated lifestyle factors in South Korea
Source: Sci Rep. 2021 Jan 28;11:2413. doi: 10.1038/s41598-021-81877-2 (PMC7844005; doi:10.1038/s41598-021-81877-2)
Supplement: Supplementary file 1 — Supplementary Information [file 41598_2021_81877_MOESM1_ESM.docx]

SUPPLEMENTARY INFORMATION

Time trends of colorectal cancer incidence and associated lifestyle factors in South Korea

Hayeong Khil^1†^, Sung Min Kim^2†^, SungEun Hong^1^, Hyeon Min Gil^3^, Eugene Cheon^3^, Dong Hoon Lee^4^, Young Ae Kim^5^* and NaNa Keum ^1,3,4^*

^1^ Department of Food Science and Biotechnology, Dongguk University Graduate School, Gyeonggi 10325, South Korea

^2^ Department of Biomedical Sciences, Seoul National University Graduate School, Seoul 03080, South Korea

^3^ Department of Food Science and Biotechnology, Dongguk University, Gyeonggi 10325, South Korea

^4^ Department of Nutrition, Harvard T.H. Chan School of Public Health, Boston, MA 02138, USA

^5^ Division of Cancer Control & Policy, National Cancer Center, Gyeonggi 10408, South Korea

† These authors contributed equally as co-ﬁrst authors.

***** Correspondence: [nak212@mail.harvard.edu](mailto:nak212@mail.harvard.edu) (N.N.K.)**;** [elkim7@gmail.com](mailto:elkim7@gmail.com) (Y.A.K.); Tel.: +1-617-432-4648 (N.N.K.)**;** Tel.: +82-31-920-2947 (Y.A.K.); Fax: +1-617-432-2435 (N.N.K.)**;** Fax: +82-31-920-2949 (Y.A.K.)

(a)


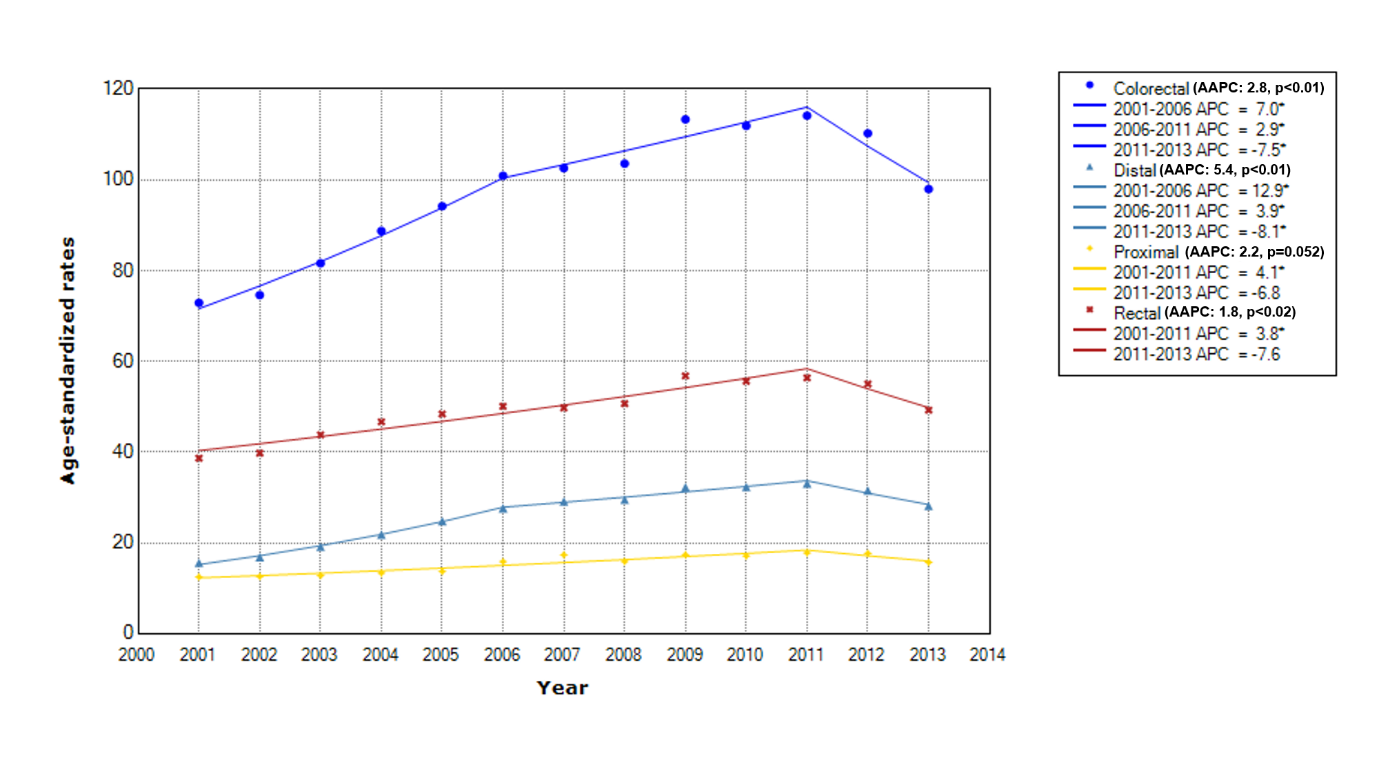


(b)


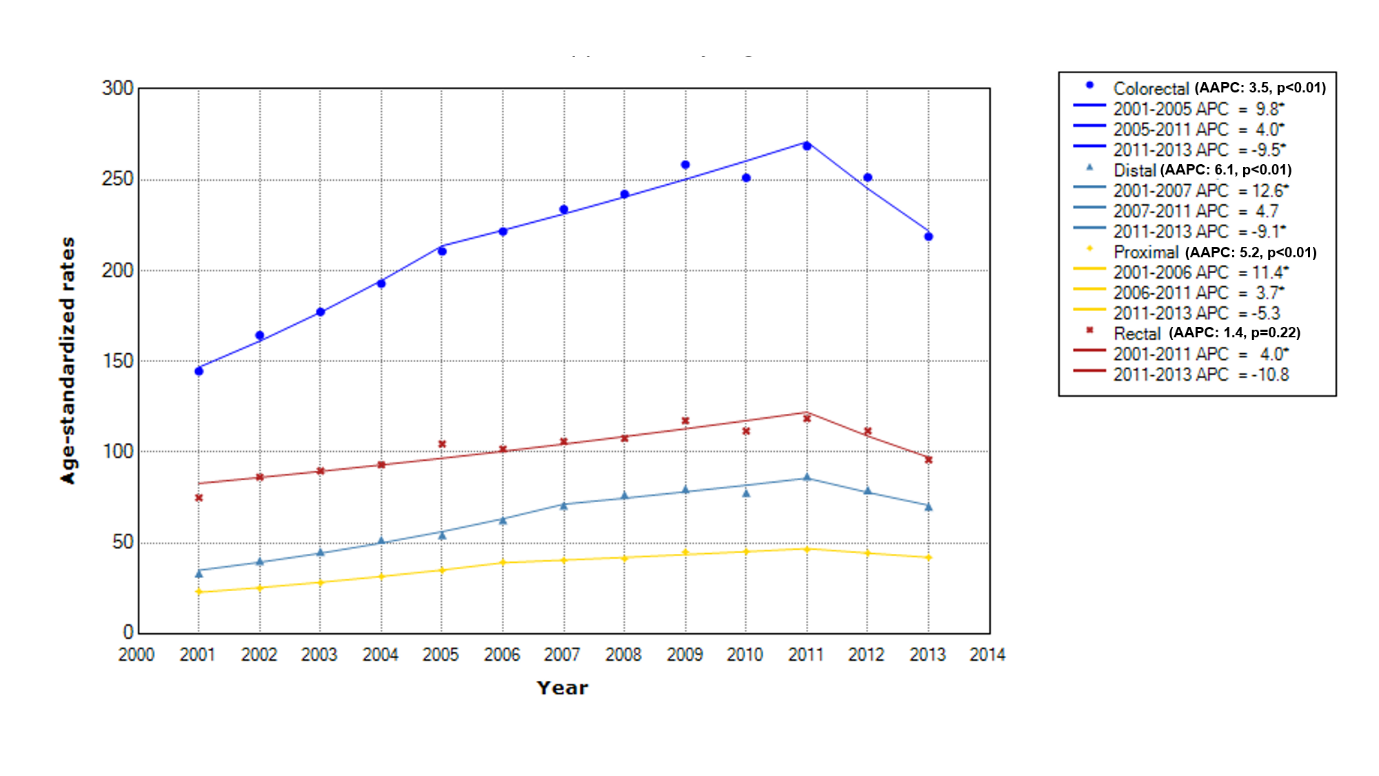


(c)


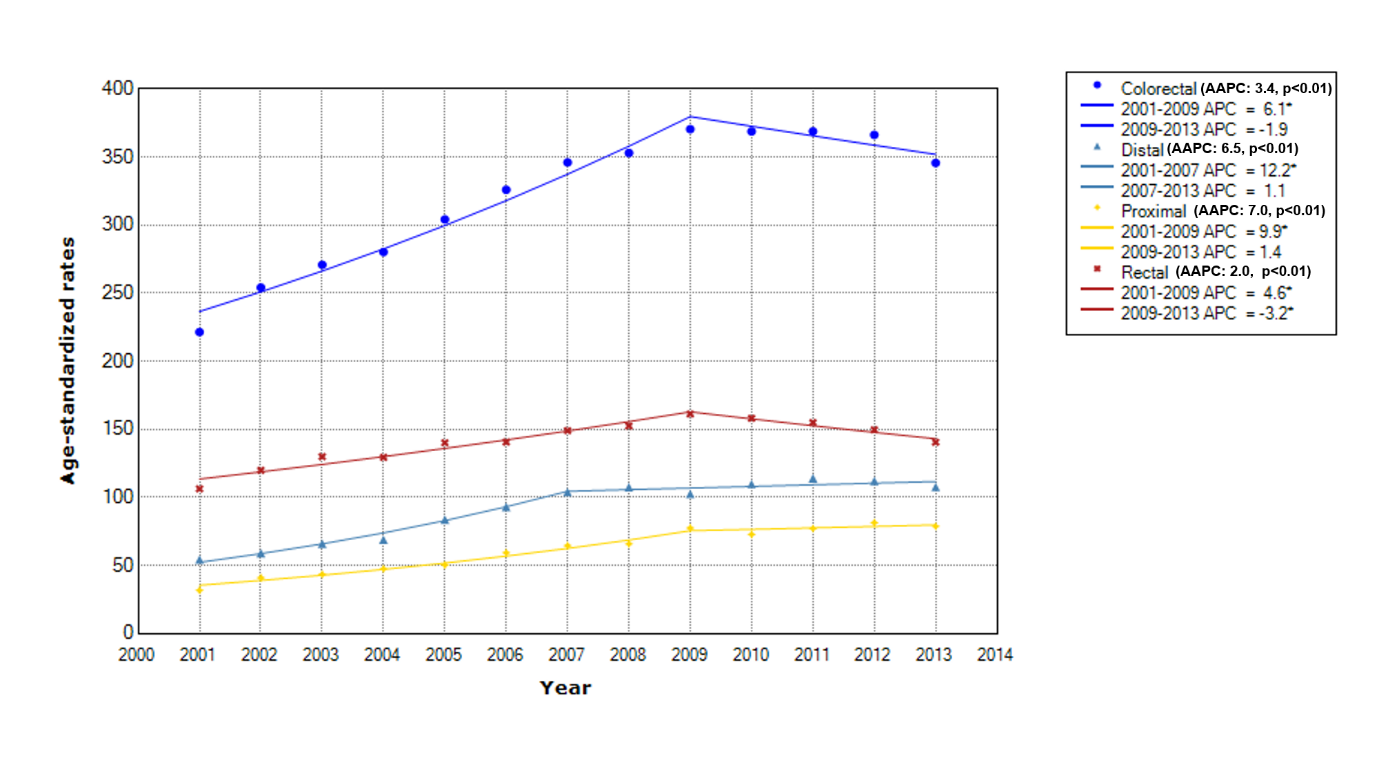


(d)


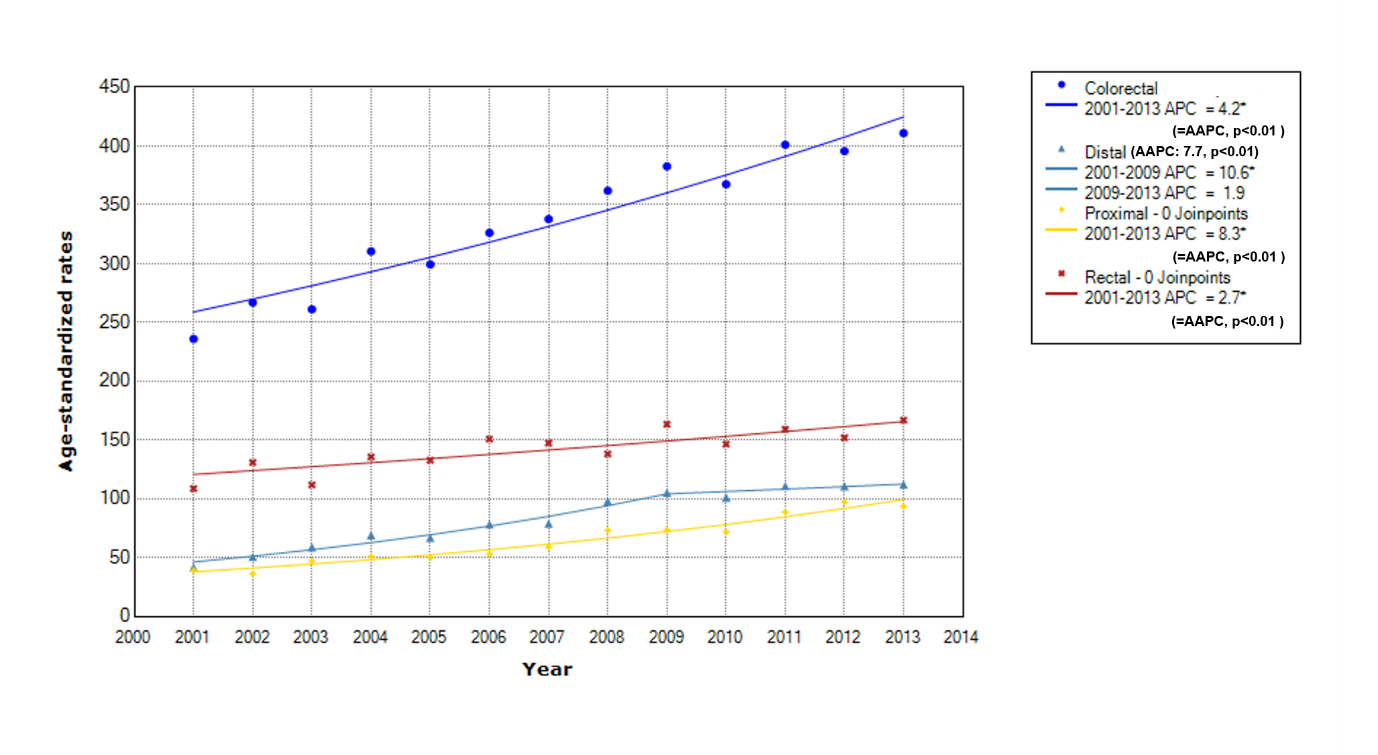


**Figure S1. Age-specific incidence rates of colorectal cancer in men.** (a) Age 50 to 59 years in men. (b) Age 60 to 69 years in men. (c) Age 70 to 79 years in men. (d) Age 80 years or more in men.

* Indicates that APC is significantly different from zero at the alpha = 0.05 level

(a)


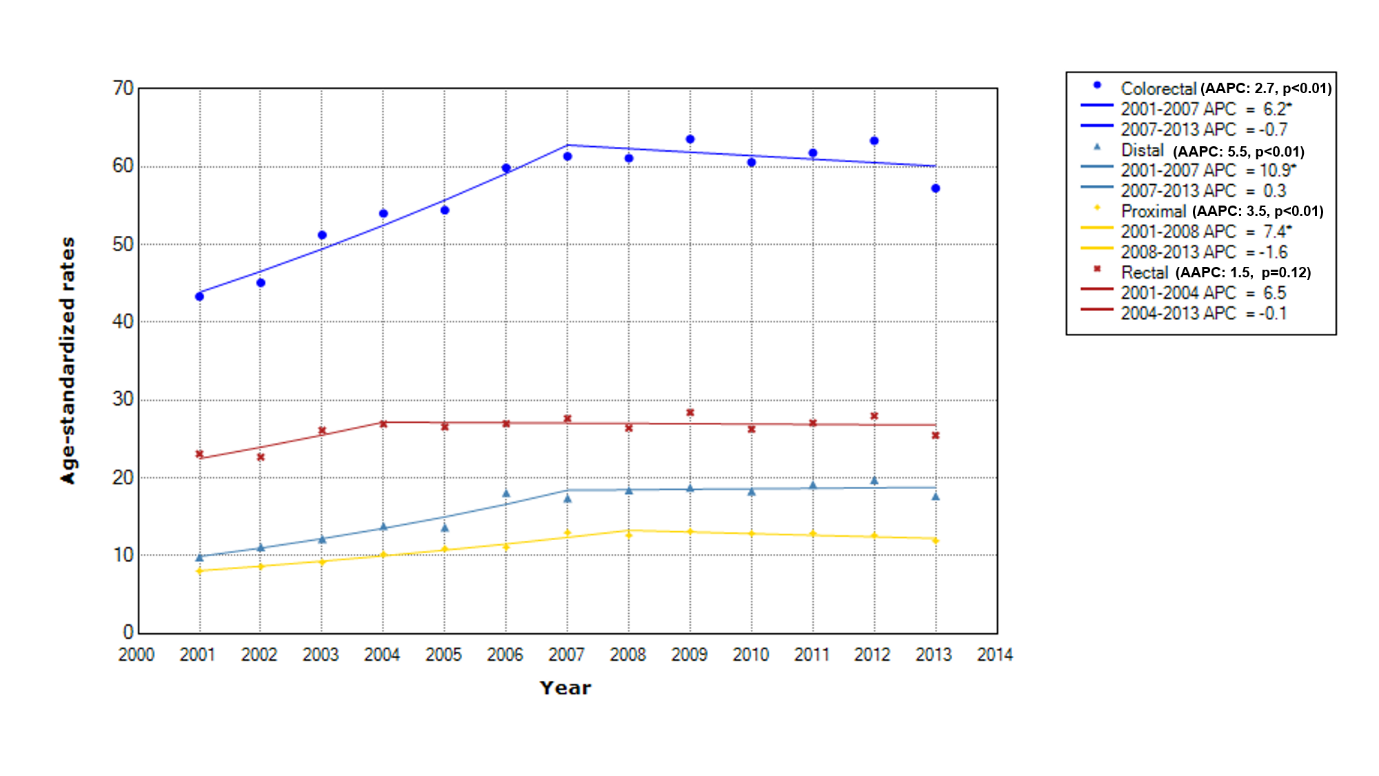


(b)


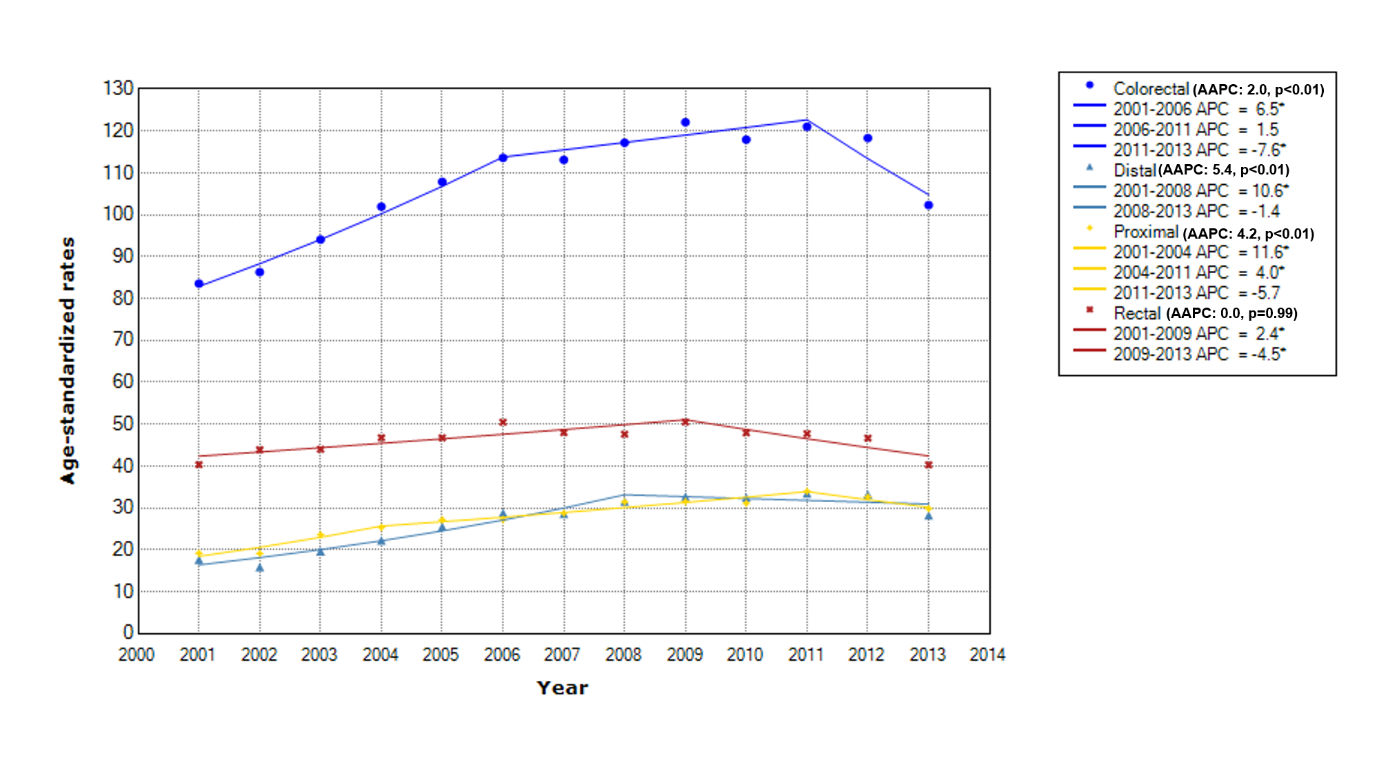


(c)


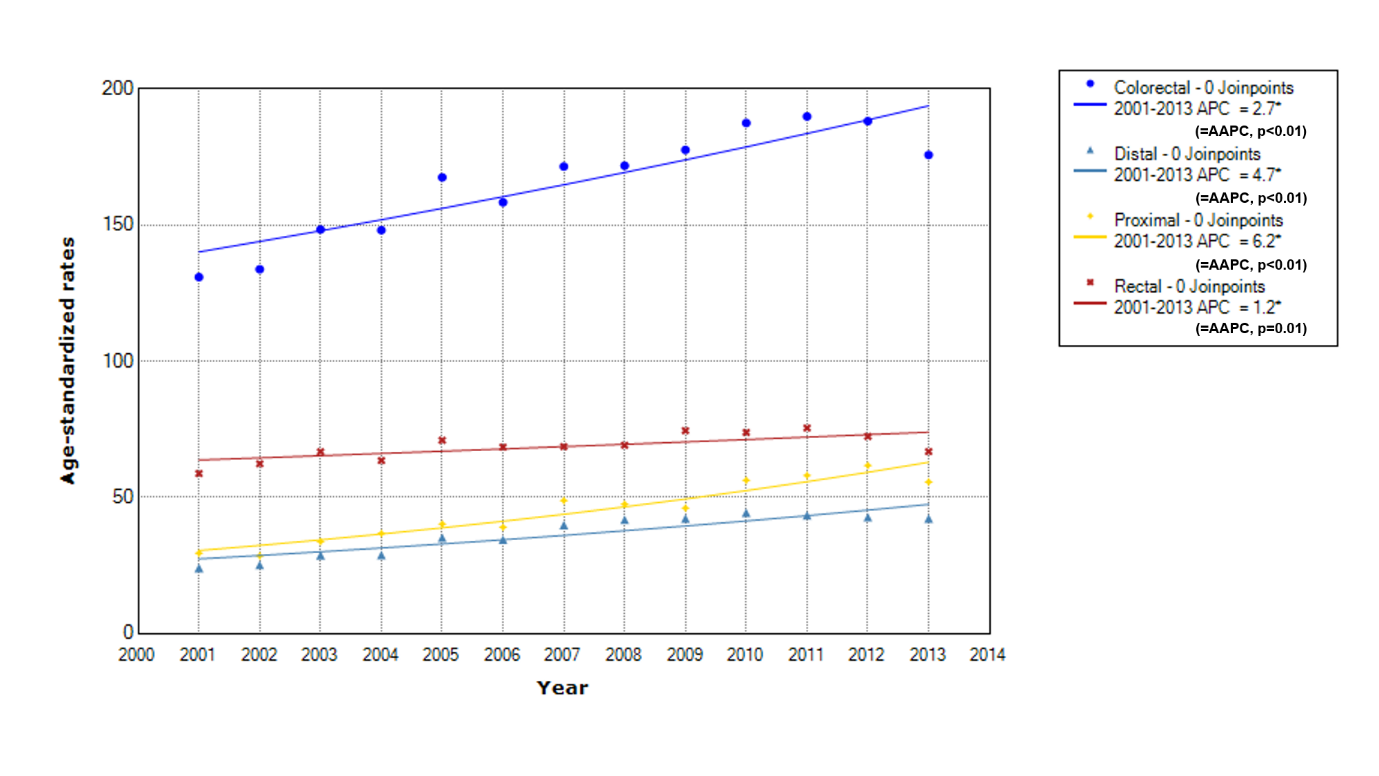


(d)


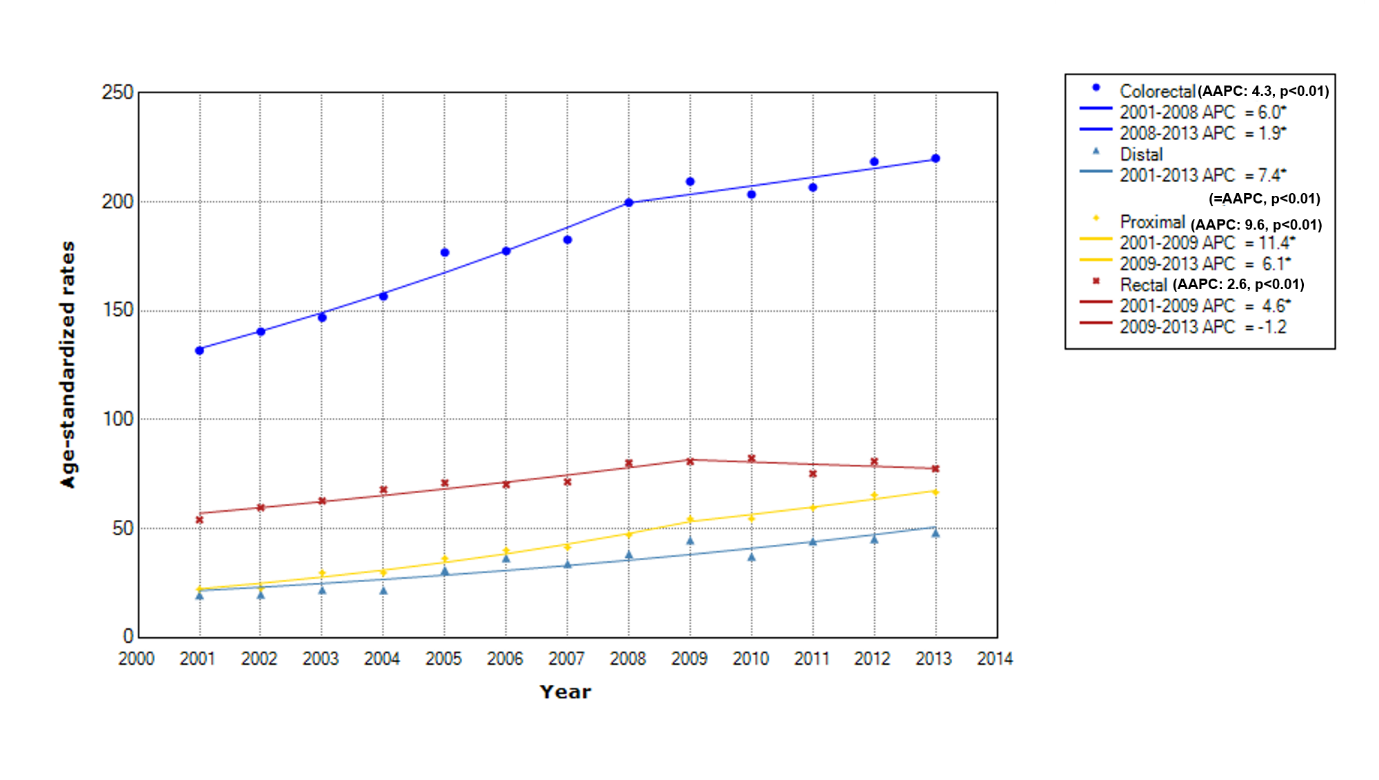


**Figure S2. Age-specific incidence rates of colorectal cancer in women.** (a) Age 50 to 59 years in women. (b) Age 60 to 69 years in women. (c) Age 70 to 79 years in women. (d) Age 80 years or more in women. * Indicates that APC is significantly different from zero at the alpha = 0.05 level
